# Pyogenic Granuloma Following Toenail Avulsion Surgery: A Case Report

**DOI:** 10.7759/cureus.114303

**Published:** 2026-08-10

**Authors:** Noopur Patel, Keisi Markaj, Michelle Gallagher

**Affiliations:** 1 Osteopathic Medicine, Michigan State University, East Lansing, USA; 2 Pediatrics, Michigan State University College of Osteopathic Medicine, East Lansing, USA

**Keywords:** nail fungus treatment, pathophysiology, pyogenic granuloma, shave biopsy, toenail

## Abstract

Pyogenic granuloma is a benign vascular proliferation that often develops following trauma or surgical procedures, particularly in acral and periungual regions. These lesions can mimic infectious or malignant processes, and their occurrence in the periungual area warrants careful evaluation due to the potential for diagnostic uncertainty. The following case discusses a 76-year-old woman who developed a pyogenic granuloma at the site of a toenail avulsion. She presented with a nonhealing, intermittently bleeding lesion on the lateral border of the left great toenail. Clinical examination revealed a friable erythematous papule beneath a hemorrhagic crust. Gross pathologic examination demonstrated a hemorrhagic, crusted, dome-shaped lesion, and the final pathologic diagnosis was pyogenic granuloma. The lesion was managed successfully with shave biopsy, electrodessication, and Monsel's solution. This case suggests that gentle removal of an overlying surface crust, when clinically appropriate, may improve assessment of the underlying lesion and facilitate accurate diagnosis. Recognition of this condition can help physicians provide timely intervention, prevent complications, and improve patient outcomes.

## Introduction

Pyogenic granulomas are acquired, benign vascular proliferations that most often arise in response to trauma, irritation, or surgical procedures. Although once thought to be infectious in origin, neither bacterial nor fungal pathogens have been proven causative; rather, mechanical and surgical triggers remain the primary factors [1]. Thus, these lesions are commonly found on acral surfaces, periungual areas, and mucous membranes, where they may present with rapid growth and frequent bleeding [2]. The lesion appears as a rapidly growing, red to purple, friable papule or polypoid mass that bleeds easily with minor trauma [2]. Pyogenic granulomas are commonly seen in children and young adults, although they may also occur in older patients. Hormonal influences appear to play a role, as pyogenic granulomas are frequently observed in pregnant women, likely related to elevated estrogen levels, and have also been reported in patients using estrogen-containing oral contraceptives [3]. Spontaneous resolution is rare, and treatment is typically required to prevent recurrence and persistent bleeding [4]. Biopsy plays a central role in confirming the diagnosis of pyogenic granuloma and distinguishing it from malignant lesions such as amelanotic melanoma [3,5]. Reported treatment modalities include curettage with cauterization, silver nitrate, and biopsy when performed as excision [3,4]. Histopathologic examination typically reveals hyperplastic capillary clusters arranged in a lobular pattern [4].

In this report, we present the case of a 76-year-old woman who developed a periungual pyogenic granuloma after toenail avulsion. Published reports of periungual pyogenic granulomas after toenail avulsion are limited, making awareness of this uncommon postoperative complication important for clinicians performing minor surgical procedures.

## Case presentation

**Figure 1:**
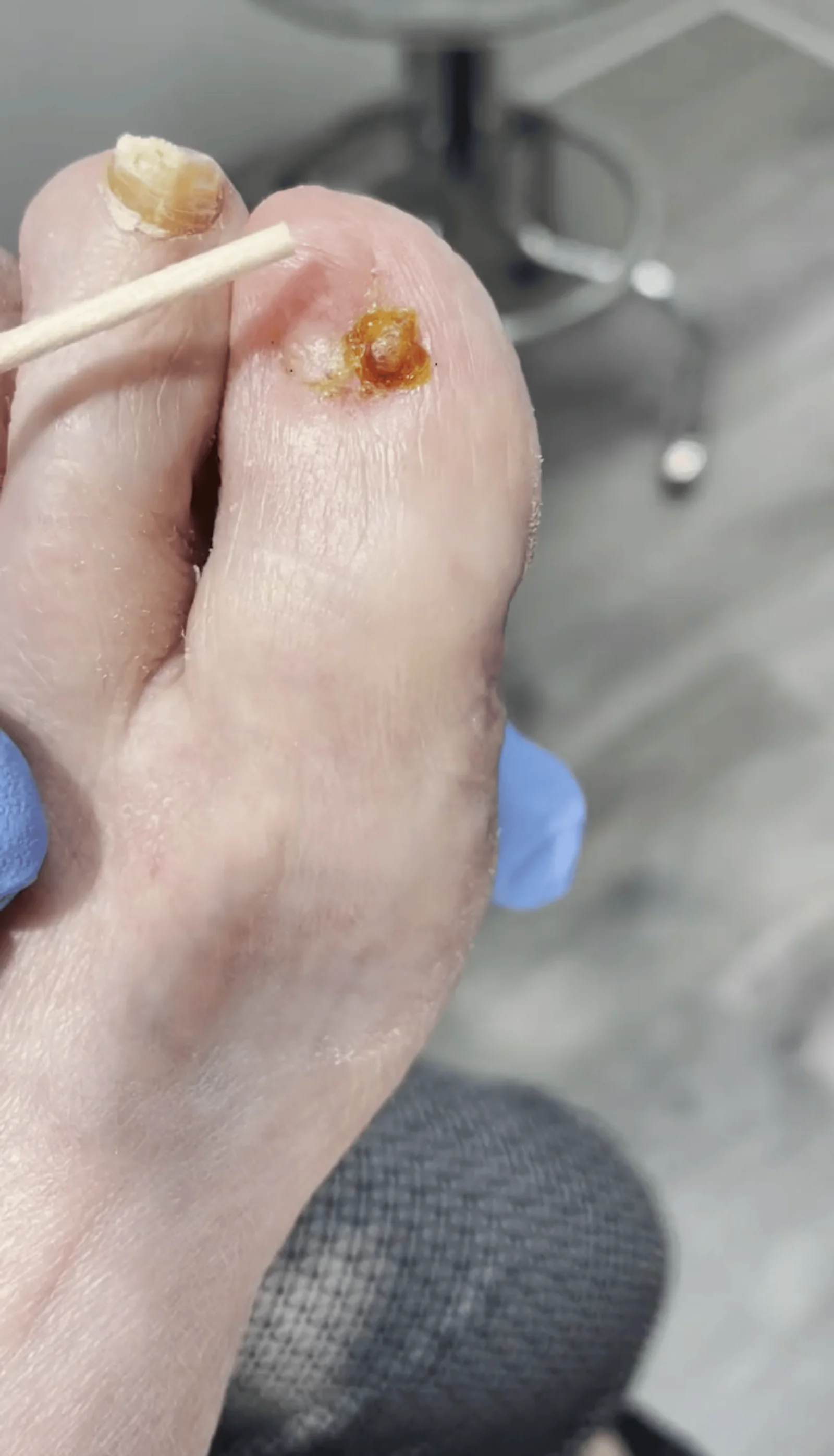
Crusted periungual lesion of the left great toe before crust removal.

**Figure 2:**
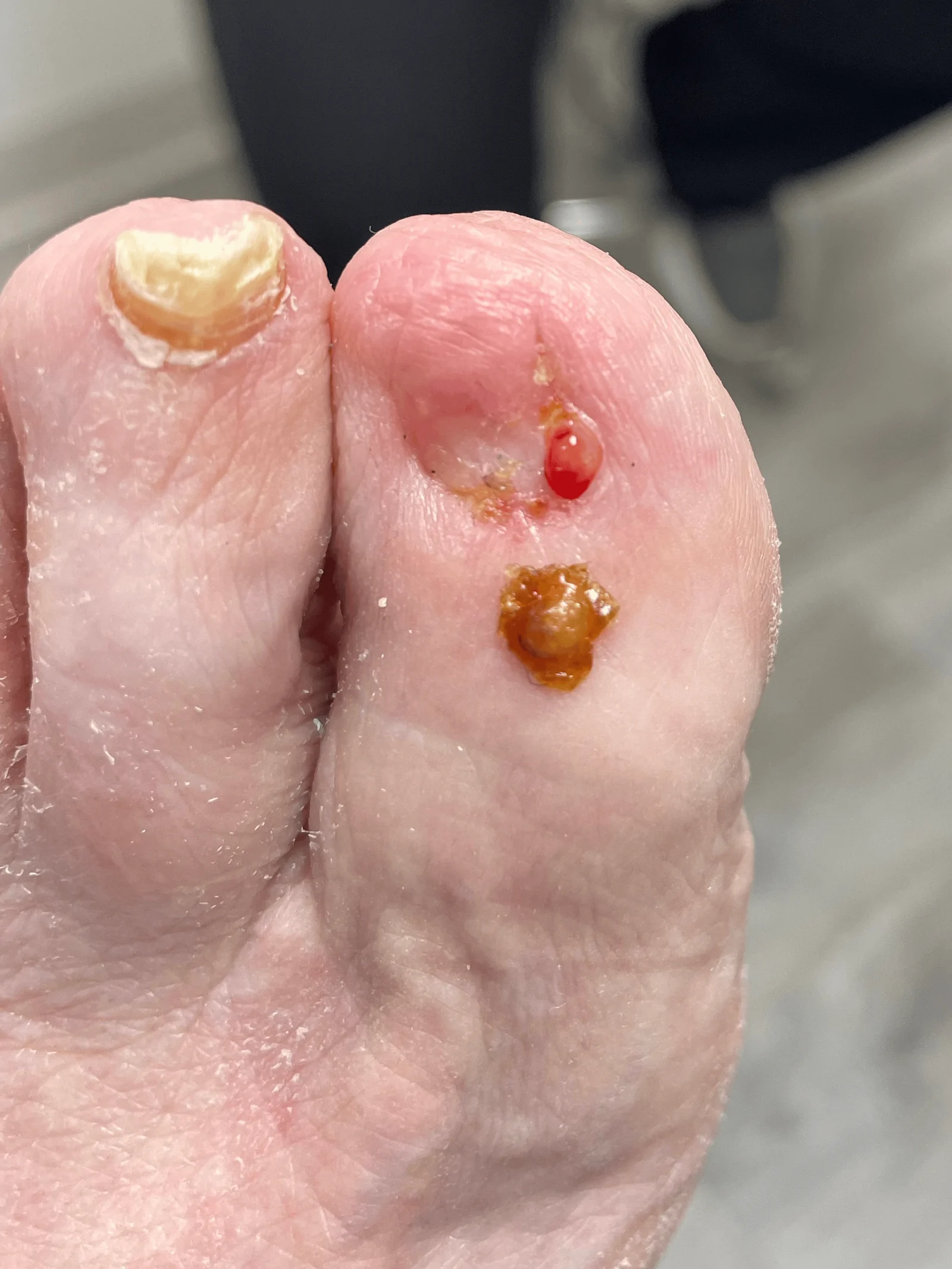
Periungual pyogenic granuloma of the left great toe after crust removal, revealing a friable, erythematous papule.

A 76-year-old woman presented with a non-healing lesion on the lateral border of the left great toenail, which developed gradually over several weeks following surgical avulsion of a mycotic nail approximately three months before presentation. She reported intermittent bleeding without associated pain or systemic symptoms. Examination revealed a 5-7 mm moist, erythematous, hemorrhagic papule on the lateral border of the left great toenail. The lesion was initially obscured by a crust, which was removed during clinical evaluation to reveal the friable papule. The lesion exhibited pinpoint bleeding with minor contact. Figure 1 and Figure 2 show the lesion before and after crust removal.

After obtaining verbal informed consent and reviewing procedural risks, including scarring, bleeding, scabbing, and incomplete removal, the left great toenail area was prepped with alcohol. Local anesthesia was achieved using approximately 0.5 mL of 1% lidocaine without epinephrine. A shave biopsy was performed to the level of the dermis using a curette. The specimen was submitted for histopathological examination with hematoxylin and eosin (H&E) staining. The wound bed was subsequently treated with electrodesiccation and curettage. Hemostasis was achieved with Monsel's solution (ferric subsulfate), and a sterile bandage was applied.

Histopathology confirmed pyogenic granuloma and excluded malignancy. Sections showed an ulcerated, polypoid lesion composed of a lobular proliferation of small, capillary-sized vessels lined by cytologically bland endothelial cells. The vessels were set in an edematous to fibromyxoid stroma with a mixed inflammatory infiltrate. The ulcerated surface was covered by fibrinopurulent exudate with associated granulation tissue and hemorrhage. No significant cytologic atypia or malignancy was identified.

Gross examination showed a tan-gray-brown, mottled, focally hemorrhagic, crusted, dome-shaped lesion involving the entire skin surface. The margin was inked green. The specimen was bisected and entirely submitted in one cassette.

The patient was provided with wound care instructions, and follow-up at three to six months, with subsequent visits as needed, demonstrated appropriate healing without evidence of recurrence.

## Discussion

In the context of existing literature, trauma and surgery are common etiologies for pyogenic granuloma. In this case, surgical avulsion of a toenail likely served as the inciting factor. The friable vascular tissue was prone to recurrent bleeding, while the overlying crust obscured the papule and delayed recognition [2]. Pyogenic granulomas are also often mistaken for wound infections because of their rapid growth, redness, and tendency to bleed [2]. For this reason, it is important to emphasize effective strategies for both diagnosis and treatment. Multiple modalities have been described, including shave excision with cauterization, cryotherapy, and laser ablation [4], although recurrence rates vary [6,7]. In our case, shave biopsy served the dual purpose of providing tissue to exclude malignant mimickers, while adjunctive electrodesiccation was used to treat the lesion base and minimize the risk of recurrence.

Consistent throughout the literature is the effectiveness of surgical excision and cauterization in the management of this condition. Surgical excision remains associated with favorable outcomes, while recurrence rates reported in the literature vary according to the treatment technique and completeness of lesion removal [4,7,8].

Despite minor differences in initial treatment strategies, clinicopathologic correlation continues to represent the cornerstone of management. It helps differentiate the unusual appearance of pyogenic granulomas from other entities, such as amelanotic melanoma or squamous cell carcinoma, which may mimic pyogenic granuloma [7].

Additionally, nail avulsion is a common podiatric procedure performed to prevent ingrown nails, infections, and other complications. This dermatologic and podiatric intersection highlights a unique overlap and underscores the importance of careful evaluation, biopsy when indicated, and timely referral to dermatology and/or podiatry.

For clinicians across primary care, dermatology, podiatry, and other procedural settings, recognizing this lesion is important, particularly when evaluating nonhealing periungual wounds following minor procedures.

## Conclusions

This case highlights a postsurgical pyogenic granuloma that was clinically obscured by an overlying crust, creating diagnostic uncertainty and potentially leading to delayed recognition. Removal of the surface crust was critical to revealing the underlying lesion, and histopathology confirmed the diagnosis. Clinicians should remain aware of this entity and its potential to present in a clinically disguised manner to facilitate timely diagnosis and management.
